# Factors Associated With No-Show to Ambulatory Tele-Video Neurology Visits

**DOI:** 10.7759/cureus.38947

**Published:** 2023-05-12

**Authors:** Aashrai Sai Venkat Gudlavalleti, John O Elliott, Rafah Asadi

**Affiliations:** 1 Neurology, OhioHealth, Columbus, USA; 2 Department of Medical Education, OhioHealth, Columbus, USA; 3 Information Analytics, OhioHealth, Columbus, USA

**Keywords:** no-show, risk factors, neurology, telehealth, teleneurology

## Abstract

Introduction

Telehealth visits (TH) have become an important pillar of healthcare delivery during the COVID pandemic. No-shows (NS) may result in delays in clinical care and in lost revenue. Understanding the factors associated with NS may help providers take measures to decrease the frequency and impact of NS in their clinics. We aim to study the demographic and clinical diagnoses associated with NS to ambulatory telehealth neurology visits.

Methods

We conducted a retrospective chart review of all telehealth video visits (THV) in our healthcare system from 1/1/2021 to 5/1/2021 (cross-sectional study). All patients at or above 18 years of age who either had a completed visit (CV) or had an NS for their neurology ambulatory THV were included. Patients having missing demographic variables and not meeting the ICD-10 primary diagnosis codes were excluded. Demographic factors and ICD-10 primary diagnosis codes were retrieved. NS and CV groups were compared using independent samples t-tests and chi-square tests as appropriate. Multivariate regression, with backward elimination, was conducted to identify pertinent variables.

Results

Our search resulted in 4,670 unique THV encounters out of which 428 (9.2%) were NS and 4,242 (90.8%) were CV. Multivariate regression with backward elimination showed that the odds of NS were higher with a self-identified non-Caucasian race OR = 1.65 (95%, CI: 1.28-2.14), possessing Medicaid insurance OR = 1.81 (95%, CI: 1.54-2.12) and with primary diagnoses of sleep disorders OR = 10.87 (95%, CI: 5.55-39.84), gait abnormalities (OR = 3.63 (95%, CI: 1.81-7.27), and back/radicular pain OR = 5.62 (95%, CI: 2.84-11.10). Being married was associated with CVs OR = 0.74 (95%, CI: 0.59-0.91) as well as primary diagnoses of multiple sclerosis OR = 0.24 (95%, CI: 0.13-0.44) and movement disorders OR = 0.41 (95%, CI: 0.25-0.68).

Conclusion

Demographic factors, such as self-identified race, insurance status, and primary neurological diagnosis codes, can be helpful to predict an NS to neurology THs. This data can be used to warn providers regarding the risk of NS.

## Introduction

Telehealth visits (TH) have become an important pillar of healthcare delivery during the COVID pandemic. By allowing social distancing and the comfort of receiving healthcare from home, TH has been widely accepted by patients [1]. TH has also been shown to be helpful in delivering care to rural areas where access to primary and specialty care is sparse [2,3].

No-shows (NS) or missed appointments without prior intimation are major problems in healthcare. They may result in delays in diagnosis and treatment, inadequate access to care for other patients, impaired quality of clinic workflow, and lost revenue [4-6].

Although the causes for NS are well known for in-person visits, there is a paucity of data for TH, especially in neurology. By understanding the factors for NS, clinicians may be able to detect individuals at high risk and take appropriate measures such as giving frequent reminders, offering social support, and considering alternative methods of providing healthcare.

In order to generate a system that can generate warnings for providers regarding the risk of NS, we believe that analysis of codified administrative data to make predictions would be a feasible option, instead of analysis of free-text provider notes. Such a system would also warn the provider regarding the risk of NS before the end of the initial encounter.

## Materials and methods

Data collection

We conducted a retrospective chart review of all telehealth video visits (THV) in our healthcare system from 1/1/2021 to 5/1/2021 (cross-sectional study). Our inclusion criteria comprised all patients at or above 18 years of age who had an ambulatory neurology THV and either had a completed visit (CV) or had an NS. Patients having missing demographic variables and not meeting the ICD-10 primary diagnosis codes for their visits were excluded. If the patient had more than one THV in the aforementioned time frame, only the first visit was included for analysis. NS was defined as missing an appointment without prior notice or notifying the clinic within 24 hours of the appointment date and time.

The ICD-10 primary diagnosis codes that were retrieved were further divided into the following diagnostic categories by the neurologist in our team (AG): dissociated and somatoform disorders, epilepsy, headache, sleep disorders, cerebrovascular disorders, myopathies/neuromuscular junction disorders, multiple sclerosis, syncope, dizziness, movement disorders, cognitive impairment, non-specific pain, gait abnormality, back or radicular pain, and neuropathies and miscellaneous. Demographics, such as age, self-reported gender, self-reported race/ethnicity, marital status, and insurance, were collected for each patient.

Statistical analysis

For comparison between NS and CV groups, independent samples t-tests were used for continuous variables, and chi-square tests were used for categorical variables. Multivariate regression, with backward elimination, was conducted adjusting for clinic location and patient zip to identify pertinent variables. Comparisons were made using a two-tailed hypothesis with significance set at p < 0.05.

## Results

**Figure 1:**
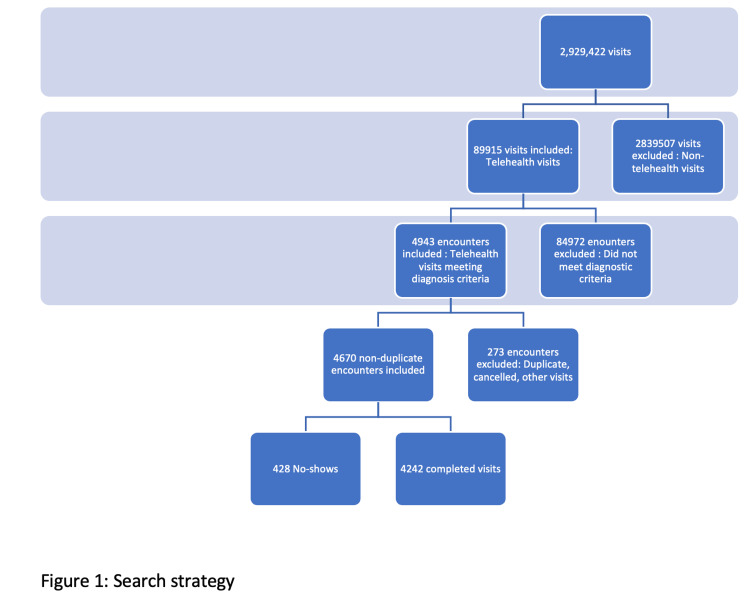
Search strategy

Our inclusion criteria resulted in 4,670 unique THV encounters out of which 428 (9.2%) were NS and 4,242 (90.8%) were CV. The search strategy is included in Figure 1, and the demographic characteristics are detailed in Table 1.

**Table 1:** Comparison of patient characteristics between NS and CV NS: no-show

	NS		
Characteristic	Yes (n = 428)	No (n = 4242)	p-value
Age, mean ± sd	46.8 ± 17.1	50.6 ± 17.6	< 0.001
Gender, % (n)			0.193
Female	65.2 (279)	68.3 (2965)	
Male	34.8 (149)	31.7 (1377)	
Race, % (n)			< 0.001
Caucasian	78.5 (335)	86.6 (3762)	
African American	15.0 (64)	9.0 (391)	
American Indian or Alaska Native	0.5 (2)	0.1 (6)	
Asian	1.4 (6)	0.9 (39)	
Native Hawaiian or other PI	0.5 (1)	0.1 (2)	
Other	0 (0)	0.1 (3)	
Declined	4.0 (17)	2.5 (107)	
Two or more races	0.5 (2)	0.7 (32)	
Marital status, % (n)			< 0.001
Single	36.4 (155)	29.0 (1259)	
Married	41.2 (176)	51.9 (2252)	
Legally separated	3.3 (14)	1.2 (53)	
Divorced	11.2 (48)	10.3 (445)	
Widowed	7.0 (30)	6.6 (286)	
Unknown	0.5 (2)	0.4 (19)	
Life partner	0.2 (1)	0.6 (25)	
Insurance payor			< 0.001
Commercial	27.1 (116)	42.9 (1864)	
Exchange	0.9 (4)	1.6 (69)	
Medicaid/managed Medicaid	31.8 (136)	17.1 (743)	
Medicare/managed Medicare	28.5 (122)	32.4 (1407)	
Unknown	9.3 (40)	5.0 (215)	
VA	2.1 (9)	0.8 (33)	
Workers comp	0.2 (1)	0.2 (10)	

There was a significant difference in age between both groups. Individuals in the NS group were significantly younger as compared to the individuals in the CV group (46.8 ± 17.1 vs. 50.6 ± 17.6; p < 0.001). There was no difference in the gender composition. Individuals identifying themselves as Caucasians were more likely to complete a visit (86.6% in the CV group vs. 78.5% in the NS group; p < 0.001). Individuals who were married were more likely to complete a visit (51.9% in the CV group vs. 41.2% in the NS group; p < 0.001). Individuals on Medicaid were more likely to NS (31.8% in the NS group vs. 17.1% in the CV group; p < 0.001).

The comparison of the primary neurological diagnoses associated with NS and CV groups is mentioned in Table 2, and the grouping of ICD codes into a specific neurological diagnosis is mentioned in Table 3.

**Table 2:** Comparison of neurological conditions between NS and CV NS: no-show

	NS		
Condition, % (n)	Yes (n = 428)	No (n = 4342)	p-value
Dissociative and somatoform disorders	0.2 (1)	0.2 (9)	0.610
Epilepsy	24.6 (106)	25.1 (1092)	0.907
Headache	23.4 (100)	26.5 (1152)	0.167
Sleep disorders	2.8 (12)	0.2 (7)	< 0.001*
TIA/stroke	5.1 (22)	4.6 (198)	0.547
Myopathies/neuromuscular junction	0.9 (4)	0.9 (41)	1.000
Syncope	1.6 (7)	0.9 (39)	0.186
Dizziness	1.9 (8)	1.5 (67)	0.543
Movement disorders	5.8 (25)	11.2 (488)	< 0.001*
Cognitive impairment	6.5 (28)	7.6 (332)	0.444
Pain, non-specific	9.3 (40)	5.9 (257)	0.008*
Gait abnormality	3.0 (13)	0.8 (33)	< 0.001*
Back/radicular pain	3.3 (14)	0.6 (27)	< 0.001*
Neuropathies	4.7 (20)	3.5 (152)	0.220
Multiple sclerosis	4.2 (18)	10.3 (448)	< 0.001*
Behavioral neurology	0.5 (2)	0 (0)	0.008*
Miscellaneous	1.6 (7)	0 (0)	< 0.001*
*p<0.05 Notes: Dissociative and somatoform disorders (F44, F45), epilepsy (G40, R56), headache (G43, G44,G50, R51), sleep disorders (G47), transient ischemic attack/stroke (G45, I63, I66, I69), myopathies/neuromuscular junction (G12, G70, G72, M21, M33), syncope (R55), dizziness (R42), movement disorders (G20, G21, G25, R25), cognitive impairment (F01. F02, F03, G30, G31, R41), pain, non-specific (M79, R20), gait abnormality (R26), back/radicular pain (M48, M51, M54), neuropathies including DM (G54, G56, G57, G58, G60, G61, G62, G63, E11), multiple sclerosis (G35, G36), behavioral neurology (F32, F41), miscellaneous (R90, M32, R29, E10, S06, G36, C71)			

**Table 3:** Diagnostic classification per ICD-10 codes

Diagnoses	ICD-10 codes
Dissociative and somatoform disorders	F44, F45
Epilepsy	G40, R56
Headache	G43, G44,G50, R51
Sleep disorders	G47
Transient ischemic attack/stroke	G45, I63, I66, I69
Myopathies/neuromuscular junction	G12, G70, G72, M21, M33
Syncope	R55
Dizziness	R42
Movement disorders	G20, G21, G25, R25
Cognitive impairment	F01, F02, F03, G30, G31, R41
Pain, non-specific	M79, R20
Gait abnormality	R26
Back/radicular pain	M48, M51, M54
Neuropathies including diabetic neuropathy	G54, G56, G57, G58, G60, G61, G62, G63, E11
Multiple sclerosis	G35, G36
Behavioral neurology	F32, F41
Miscellaneous	R90, M32, R29, E10, S06, G36, C71

Univariate analysis showed that individuals with a primary diagnosis of sleep disorders, gait abnormalities, back/radicular pain, non-specific pain, and behavioral neurology were more likely to NS. Patients carrying a primary diagnosis of movement disorders and multiple sclerosis were more likely to complete a visit.

Patients not fitting into these categories were grouped into miscellaneous disorders and were also more likely to NS. However since there was only one case for each diagnostic code in the miscellaneous group, it was not included in further discussion.

Table 4 shows the multivariate regression model with backward elimination. When adjusted for clinic locations across the health system and patient location by zip code, the attributed variance increased from 8.3% to 11.3%.

**Table 4:** Multivariate logistic regression model NS: no-show

Variable	NS (yes vs. no)		
Reduced model	Odds ratio	95% CI	p-value
Race: non-Caucasian vs. Caucasian	1.62	1.26-2.09	< 0.001
Marital status: married (yes vs. no)	0.74	0.60-0.91	0.005
Medicaid (yes vs. no)	1.71	1.46-1.99	< 0.001
Sleep disorders	22.16	8.50-57.70	< 0.001
Movement disorders	0.60	0.39-0.92	0.018
Pain, non-specific	1.62	1.13-2.31	0.008
Gait abnormality	4.09	2.10-7.99	< 0.001
Back/radicular pain	5.64	2.89-11.01	< 0.001
Multiple sclerosis	0.47	0.29-0.76	0.002
Notes: full model R^2 ^= 8.3%			

The final multivariate model after adjusting for clinic locations and patient zip codes is shown in Table 5.

**Table 5:** Multivariate logistic regression model adjusted for patient zip code and clinic location NS: no-show

Variable	NS (yes vs. no)		
Reduced model	Odds ratio	95% CI	p-value
Race: non-Caucasian vs. Caucasian	1.65	1.28-2.14	0.002
Marital status: married (yes vs. no)	0.74	0.59-0.91	0.005
Medicaid (yes vs. no)	1.81	1.54-2.12	< 0.001
Sleep disorders	10.87	5.55-39.84	< 0.001
Movement disorders	0.41	0.25-0.68	< 0.001
Gait abnormality	3.63	1.81-7.27	< 0.001
Back/radicular pain	5.62	2.84-11.10	< 0.001
Multiple sclerosis	0.24	0.13-0.44	< 0.001
Notes: Controlling for clinic location, appointment time (AM/PM), and patient zip code. Full model R^2 ^= 11.3%			

The odds of NS were higher with a self-identified non-Caucasian race, possessing Medicaid insurance, and with primary diagnoses of sleep disorders, gait abnormalities, and back/radicular pain. Primary diagnoses of multiple sclerosis and movement disorders were associated with higher odds of completed visits.

## Discussion

During the initial stages of the COVID-19 pandemic, in-person healthcare visits declined by 60%, and the use of THs increased [7]. Since the pandemic, telehealth flexibilities have been continuously extended and have helped in improving access to virtual healthcare [8]. THs have been shown to meet patient and provider expectations as compared to in-person visits [3,9]. Given the recent increase in its use, telehealth use may expand in the future and may become an integral part of delivering healthcare. NSs pose a risk in delivering quality care, having an efficient clinic workflow, and optimizing the cost of care. The data regarding NSs in telemedicine, especially in neurology, is sparse. Our study attempted to address this knowledge gap in the field of THV in neurology.

Our study highlights important predictors of NS in THVs which may be utilized to inform the provider regarding patients who are at the highest risk of NS.

NS rates have been shown to vary widely depending on the type of study, its location, and the field of medicine being studied. A study by Elkhider et al. regarding the predictors of NS in neurology clinics showed an NS rate of 16% [4]. However, Golomb et al. demonstrated a much higher NS rate in pediatric neurology clinics, around 25% [10]. In a study by Adelson et al., the NS rates were found to be as high as 47% in some cases [11]. Our study showed a much lower NS rate of 9.2%. Since the aforementioned studies were in-person visits rather than THV, it is possible that our NS rate was reflective of the advantages of THV. A study by Drerup et al. supported this hypothesis. The authors showed that NS rates for televisits during the pandemic were significantly lower than NS rates for in-office visits prior to the pandemic (7.5% for THs vs. 29.8% for in-office visits prior to the pandemic; p < 0.001) [1]. Our study showed a similar NS rate for THV (9.2%).

There were multiple associations between NS, demographic factors, and ICD-10 diagnostic codes. However, only the variables achieving significance on multivariate regression were considered important and are discussed below.

Prior studies have demonstrated higher rates of NS in satellite clinics rather than in the primary location. A study of 22,759 scheduled clinic visits at the University of Kentucky demonstrated that NSs were higher in their satellite clinics as compared to the university-based location (25% vs. 19%; p < 0.0001) [12]. Patient zip codes have also been shown to be a factor in NS. In a study of NS rates in an academic pediatric otolaryngology practice, the authors reported the highest NS rates in the zip codes with the lowest median income [6]. In our study, adjusting for patient zip codes and clinic locations resulted in an increase in variance in our multivariate regression model (R2 of 8.3% without adjusting for the variables vs. R2 of 11.3% after adjusting for the variables, Table 5).

The following variables were significant in the adjusted multivariate regression model: self-identified Caucasian background, Medicaid insurance, sleep disorders, movement disorders, gait abnormalities, back/radicular pain, and multiple sclerosis.

Self-identified race

Individuals identifying themselves as Caucasian were more likely to complete a visit. Race and ethnicity are closely tied with socioeconomic status in the United States. The report of the APA task force on socioeconomic status published in 2007 showed higher median wealth for White individuals as compared to Black or Hispanic individuals [13]. Darrat et al. demonstrated socioeconomic disparities in the utilization of telehealth during the COVID pandemic. Individuals in the lowest median income quartile had lower odds of completing a virtual visit [14]. Hence, our findings may reflect this income and resource utilization gap. Further studies are needed to explore these associations.

Medicaid insurance

Our study showed that the odds of NS are higher in individuals with Medicaid insurance 1.81 (1.54-2.12), p<0.001). These findings are consistent with numerous other studies which have demonstrated that individuals on Medicaid insurance are likely to NS to their clinic appointments [4,14,15]. This also suggests that individuals with low income are more likely to NS.

These findings suggest lower utilization of healthcare among individuals in the low-income group (LIG). Since the start of the COVID-19 pandemic, THVs have been well reimbursed. Although the ease of access to healthcare improved with the pandemic, our findings suggest that given the costs, the benefit of THVs may not have reached individuals in the LIG. The study by Darrat et al. showed that the individuals within the lowest income quartile were less likely to complete a virtual visit but were more likely to complete a telephone visit. Individuals on Medicaid were also more likely to complete a telephone visit [14]. The higher reimbursement and cost of THV, the cost of an electronic device that facilitates THVs, and the cost of Internet access are likely barriers for individuals in LIG to be able to access healthcare via video.

Visit diagnoses

Sleep Disorders

In our study, sleep disorders had the highest odds of NS 10.87 (5.55-39.84). A retrospective review performed by David et al. found a high NS rate in sleep disorder clinics of 20%; i.e., one in five individuals was likely to NS [16]. An intuitive reason would be the inability to attend the clinic due to excessive daytime fatigue; however, other problems, such as cognitive impairment and inattention, cannot be ruled out. More research is needed in this field to evaluate the reason for NS.

Movement Disorders

Patients with movement disorders were more likely to complete their THV. Our findings are consistent with prior studies of NS rates in patients with and without Parkinson’s disease [17]. The authors found that the overall NS rate was lower for patients with Parkinson’s disease as compared to those with other neurological disorders (3% vs. 7.4%, respectively). Elkhider et al. also demonstrated lower odds for NS among patients with movement disorders [4]. One reason is that these patients require longitudinal care and are, thus, more likely to follow up.

Gait Abnormality

Individuals with gait disorders were more likely to NS to their THVs. Given that mobility might be an issue, one would expect a higher acceptance of THV among patients with gait problems. Gait assessments via telehealth have been shown to have similar inter-rater reliability as compared to in-clinic assessments [18]. Our literature search did not find any studies which looked into barriers to adherence to clinic visits in patients with gait disorders. Given that gait can be monitored remotely via smartphone applications, and given that the reliability of gait assessments over telehealth appears to be similar to in-clinic assessments, telehealth may be a great tool to provide care to individuals with gait disorders. Hence, further studies are required to assess barriers to the acceptance of THV in patients with gait disorders.

Back/Radicular Pain

Individuals with back or radicular pain also had higher odds of NS. Chronic pain has also been associated with high NS rates in other studies. Odonkor et al. found an NS rate of approximately one in four or 25% in a longitudinal prospective study in an academic pain center. The rate of NS was also found to be higher in individuals complaining of low back pain (OR = 4.1, 95% CI 1.7-10, p < 0.001) [19]. A possible reason for higher NS in pain may be due to the fact that pain symptoms may require multiple visits to different physicians, which may increase the risk of NS.

Multiple Sclerosis

Individuals with multiple sclerosis were less likely to NS. Similar to movement disorders, multiple sclerosis is a chronic condition that needs long-term follow-up. Hence, individuals are more likely to be adherent to their visits. Multiple sclerosis has been shown to be associated with lower odds of NS in other studies as well [4].

Our study showed that certain demographic variables, such as self-identified race/ethnicity and insurance status, as well as certain diagnoses, such as sleep disorders, back/radicular pain, and gait abnormalities, are associated with a higher risk of NS to neurology THs. Diagnoses, such as movement disorders and multiple sclerosis, are associated with a lower risk of NS. The reasons for a higher risk of NS with some neurological disorders and a lower risk of NS with others are still unclear and demand further research. Studies also need to look into ways of improving access and acceptance to THVs for the LIG since the utilization of this resource in the LIG remains low. Public health programs, such as financial assistance and discounted rates for smartphones and the Internet, may help improve access to THVs.

Another finding of our study was that most common neurological disorders, such as migraine, epilepsy, and cerebrovascular diseases, were amenable to THVs and were not linked to a higher risk of NS. The feasibility of telehealth in neurology has been demonstrated in multiple studies [3,20]. Our study supports the use of THVs in most neurological disorders, at least from a clinic attendance standpoint.

A major strength of our study is the sample size of 4670 unique encounters. Although we did not conduct a prespecified sample size calculation, we conducted a brief post hoc sample size calculation using STATA statistical software (StataCorp, Texas, USA) for the variables age and sleep disorder. (Sleep disorder was the significant variable with the least difference in proportions between the groups, i.e., 2.6%). Our sample size far exceeds the sample size required to find a difference in age of four years with a standard deviation of 17 years, with 90% power (the sample size required is 381 patients in each group). Our study sample size also far exceeds the sample size required to find a difference of 2.6% between groups with 80% power (the sample size required is 342 per group). Hence, it can be assumed that our study was sufficiently powered (80%) to find a difference of at least 2.6% between variables.

The findings of our study can help facilitate the creation of a system that can predict the risk of NS for a patient with a neurological disorder using administrative data. This can be used to inform the provider beforehand regarding the risk of NS. Such predictions can be used in clinical practice in multiple ways. The provider may consider alternate methods of care, such as telephone visits or in-person care. The provider or the healthcare system may schedule patients in a way that is likely to result in the least number of NSs in a day. Lastly, the provider or the healthcare system may consider targeted intervention for high-risk individuals such as frequent telephone or electronic reminders, focused discussion groups, and considering providing smart devices and Internet access at subsidized rates. The developing field of artificial intelligence may aid in predicting NSs and optimizing scheduling in the future [21].

Our study has several limitations. The records were assessed from only one health system in central Ohio; hence, its generalizability is limited. We only looked at the data that could be obtained from the administrative database. Hence, there may be multiple other factors that may contribute to NS and may have been missed. One of them is the time lag between scheduling the appointment and the date of the visit, which has been shown to be linked to the risk of NS [11]. We had to exclude individuals with missing data, which may have led to selection bias. Another limitation of the study is that we used primary ICD-10 diagnosis codes for analysis and did not take into account other diagnoses. For example, if the primary diagnosis code was for gait disorder, but the patient also had secondary diagnosis codes for stroke or Parkinson's disease, the patient was classified as having a gait disorder. This may have led to misclassification bias but is likely a limitation of using administrative data that cannot be completely eliminated. However, the aforementioned selection and misclassification biases would likely be non-differential (would not differ between NS and CV groups) and, hence, should not affect the internal validity of the study, although they may limit the generalizability.

## Conclusions

In conclusion, our study highlights the demographic and clinical factors associated with NS to ambulatory tele neurology visits. This data can help administrators and clinical providers to schedule televisits for individuals who are more likely to complete them and consider alternate methods of delivering care to individuals who are at a higher risk of NSs. The variables that we considered in our final multivariable logistic regression model accounted for only 11 % of the variance. We believe that this is because of the limited number of variables that could be analyzed in our administrative dataset. We believe that with the help of machine learning, a greater number of variables can be analyzed and incorporated into the model, thus improving its accuracy.

Further research is needed to look into other factors predicting NS to neurology THVs and to create accurate prediction tools to reduce NSs in neurology. This will lead to improvement in the quality of patient care and the efficiency of ambulatory neurology practice.
